# Characterization of miRNAs in Cultured Atlantic Salmon Head Kidney Monocyte-Like and Macrophage-Like Cells

**DOI:** 10.3390/ijms21113989

**Published:** 2020-06-02

**Authors:** Nicole C. Smith, Sherri L. Christian, Nardos T. Woldemariam, Kathy A. Clow, Matthew L. Rise, Rune Andreassen

**Affiliations:** 1 Department of Ocean Sciences, Memorial University of Newfoundland, 0 Marine Lab Road, St. John’s, NL A1C 5S7, Canada; g54ncs@mun.ca (N.C.S.); kclow@mun.ca (K.A.C.); 2 Department of Biochemistry, Memorial University of Newfoundland, 232 Elizabeth Ave, St. John’s, NL A1B 3X9, Canada; sherri.christian@mun.ca; 3 Department of Life Sciences and Health, Faculty of Health Sciences, OsloMet–Oslo Metropolitan University, N-0130 Oslo, Norway; nate@oslomet.no (N.T.W.); rune.andreassen@oslomet.no (R.A.)

**Keywords:** Atlantic salmon, macrophages, miRNA, head kidney culture, sequencing, cell differentiation

## Abstract

Macrophages are among the first cells to respond to infection and disease. While microRNAs (miRNAs) are involved in the process of monocyte-to-macrophage differentiation in mammals, less is known in teleost fish. Here, Atlantic salmon head kidney leukocytes (HKLs) were used to study the expression of miRNAs in response to in vitro culture. The morphological analysis of cultures showed predominantly monocyte-like cells on Day 1 and macrophage-like cells on Day 5, suggesting that the HKLs had differentiated from monocytes to macrophages. Day 5 HKLs also contained a higher percentage of phagocytic cells. Small RNA sequencing and qPCR analysis were applied to examine the miRNA diversity and expression. There were 370 known mature Atlantic salmon miRNAs in HKLs. Twenty-two miRNAs (15 families) were downregulated while 44 miRNAs (25 families) were upregulated on Day 5 vs. Day 1. Mammalian orthologs of many of the differentially expressed (DE) miRNAs are known to regulate macrophage activation and differentiation, while the teleost-specific miR-2188, miR-462 and miR-731 were also DE and are associated with immune responses in fish. In silico predictions identified several putative target genes of qPCR-validated miRNAs associated with vertebrate macrophage differentiation. This study identified Atlantic salmon miRNAs likely to influence macrophage differentiation, providing important knowledge for future functional studies.

## 1. Introduction

Macrophages are some of the first cells that respond to infection and disease. They are critical in mounting and resolving an immune response during tissue injury and/or pathogen infection [1]. Macrophages are derived from hematopoietic progenitors which differentiate from their precursor cells, monocytes [2]. Macrophage differentiation is tightly controlled via a multitude of cytokines, growth factors and transcription factors such as colony-stimulating factor 1 (CSF-1) and the transcription factors PU.1 and Runx1 [2,3]. In mammals, two distinct subsets of macrophages have been described: M1 (classically activated macrophages) and M2 (alternatively activated macrophages) [4]. M1 macrophages are induced by cytokines primarily secreted by Th1 cells, including IFN-γ and TNF-α, and are involved in the inflammatory response, producing reactive oxygen species (ROS) and inflammatory cytokines. M2 macrophages are induced by cytokines including IL-4, IL-13 and TGF-β and are involved in wound healing, repair and immune suppression [4,5,6]. M2 macrophages can be further divided into various subtypes (M2a, M2b and M2c), based on their activation and function [6]. Much of our knowledge of fish macrophage differentiation and activation comes from zebrafish (*Danio rerio*), ginbuna carp (*Carassius auratus langsdorfii*) and goldfish (*C. auratus*, L.) models [7,8]. Important factors in mammalian macrophage differentiation, such as CSF-1, CSF-1R, and Toll-like receptors, as well as factors involved in M1/M2 macrophage activation, such as TNF-α, interferons, IL-4/13 and arginase, have been identified and characterized in fish studies (reviewed in [7,9,10]). While our knowledge of fish macrophage biology is expanding, macrophage differentiation and activation across all teleost species, including the Atlantic salmon, an economically important farmed fish, remain to be adequately described.

MicroRNAs (miRNAs) are short, non-coding RNAs (ncRNAs) that play roles in controlling many biological processes through post-transcriptional regulation of gene expression [11,12,13]. Following transcription, the primary miRNA transcript is cleaved by Drosha into precursor miRNAs, followed by exportation of the precursor miRNA out of the nucleus by exportin 5. The precursor miRNA is then processed further by Dicer to produce mature miRNAs (5p or 3p) that are 20–24 nucleotides in length, which are then incorporated into the miRNA-induced silencing complex (miRISC) [12,13]. As part of the miRISC, the miRNA can then bind to its partially complementary sequence, usually located in the 3′-untranslated region (3′-UTR), of its target mRNA, leading to mRNA degradation or the prevention of translation. miRNAs regulate several biological processes including, but not limited to, cell differentiation, cell development, apoptosis and immune response [14,15].

Mammalian studies have demonstrated that several miRNAs mediate the activation and differentiation of macrophages (reviewed in [16,17]). For example, a study in murine bone marrow-derived macrophages found that the expression levels of 109 miRNAs were altered between M1 and M2 conditions [18]. Functional studies have demonstrated that some miRNAs (e.g., miR-9, miR-127, miR-155 and miR-125b) promote the activation of M1 macrophages and the pro-inflammatory response, while other miRNAs (e.g., miR-124, miR-34a, let-7c, miR-132, miR-146a and miR-125a-5p) promote the activation of M2 macrophages and the anti-inflammatory response [17]. Several miRNAs are also capable of regulating myeloid cell development, including monocyte-to-macrophage differentiation. For instance, miR-15, miR-20a and miR-106a target the 3′-UTR of Runx1, a transcription factor that controls the expression of colony-stimulating factor-1R (CSF-1R) which promotes differentiation and maturation to the monocyte lineage [19]. Small RNA profiling has identified miRNAs involved in the immune response of fish, such as differential miRNA expression in immune tissues following pathogen exposure (reviewed in [20]). However, few studies have examined miRNAs involved in fish macrophage differentiation, activation and/or function [21,22,23,24,25].

In fish, a population of adherent leukocytes, consisting of heterogenous cells including monocytes and macrophages, can be isolated and cultured from the anterior (or head) kidney (HK), the main hematopoietic organ in fish, equivalent to the mammalian bone marrow [26,27,28]. While these cells are frequently used in fish immunological studies ([26,29,30,31,32], to name a few), their transcriptome and function during culture remain poorly characterized. Our results show that the morphology and phagocytic ability of adherent head kidney leukocytes (HKLs) change during culture time. To more thoroughly characterize this change at the molecular level, we have examined the miRNA expression profile in adherent HKLs from Atlantic salmon as they differentiate during culture time.

## 2. Results

### 2.1. Influence of Culture Time on the Morphology, Phagocytic Ability, Reactive Oxygen Species (ROS) Production and Macrophage Markers in Atlantic Salmon Adherent HKLs

**Figure 1:**
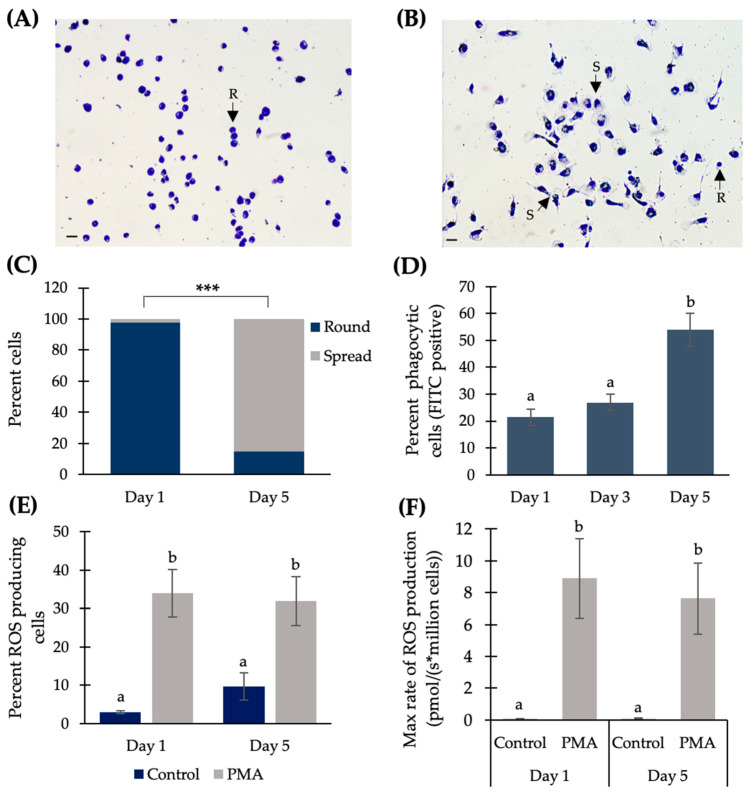
Influence of culture time on the morphology and function of Atlantic salmon adherent head kidney leukocytes (HKLs). Representative images of Giemsa-stained HKLs cultured for (**A**) 1 day and (**B**) 5 days. Arrows indicate spread cells (S; cells with pseudopodia present) and non-spread, round cells (R; cells with no pseudopodia present). Scale bar on the bottom left of panel (**A**) and panel (**B**) is equal to 20 μm. (**C**) Mean percentage of round vs. spread cells in Day 1 and Day 5 cultures, where *** indicates a significant difference of *p* < 0.0001 by chi-square test. (**D**) Percentage of phagocytic HKLs on Day 1 and Day 5 of culture. (**E**) Percentage of HKLs producing reactive oxygen species (ROS), as determined by flow cytometry. (**F**) Maximum rate of ROS production (pmol/(s*million cells)), as determined by Oroboros respirometry. Day 1 control value: 0.089 pmol/(s*million cells); Day 5 control value: 0.105 pmol/(s*million cells). Data shown as the mean +/− SE; different lowercase letters indicate a significant difference of *p* < 0.05 as determined by a repeated measures one-way ANOVA for phagocytosis data and a paired Student’s *T*-test for ROS data, *n* = 4. PMA: phorbol myristate acetate.

On Day 1 and Day 5 of culture, Atlantic salmon adherent HKLs were stained with Giemsa and imaged to observe cell morphology. On Day 1, the majority of cells were round (R) with no pseudopodia (i.e., non-spread; Figure 1A), while on Day 5, the majority of the cells had pseudopodia present (i.e., spread (S); Figure 1B). On Day 1, the cell population consisted of 97.8% round, non-spread cells (range of 97.1–98.8%) and 2.2% spread cells (range of 1.2–2.9%) while on Day 5, 14.9% of the cells were round and non-spread (range of 10.5–21.1%) and 85.1% of the cells were spread (range of 79.0–89.5%) (Figure 1C). There was a significant change in the proportion of round and spread cells on Day 1 vs. the proportion of round and spread cells on Day 5 (*p* < 0.0001).

To determine how the phagocytic ability of HKLs changes from Day 1 to Day 5 of culture, cells were incubated with fluorescent (FITC) beads and, 24 h later, FITC fluorescence was analyzed via flow cytometry. On Day 1, 21.4 ± 3.1% (SE) of the cells were phagocytic; on Day 3, 26.9 ± 2.9% of the cells were phagocytic; and on Day 5, 53.9 ± 6.1% of the cells were phagocytic (Figure 1D). There was a significant increase in the percentage of phagocytic cells in cells cultured for 5 days compared to cells cultured for 1 day (Tukey’s post-hoc *p* = 0.048) and 3 days (Tukey’s post-hoc *p* = 0.015). There was no significant difference in the percentage of phagocytic cells in Day 1 and Day 3 samples (*p* = 0.442).

To determine whether the percentage of HKLs producing ROS changed during culture duration, on Day 1 and Day 5, cells were treated with phorbol myristate acetate (PMA) to stimulate ROS production, or dimethylsulfoxide (DMSO) as a negative control, and analyzed via flow cytometry (Figure 1E). On Day 1, 2.95 ± 0.39% (SE) of the DMSO negative control cells produced ROS, while 33.98 ± 6.09% of the PMA-stimulated cells produced ROS. On Day 5, 9.73 ± 3.52% of the DMSO negative control cells produced ROS, while 32.0 ± 6.35% of the PMA-stimulated cells produced ROS (Figure 1E). There was a significant increase in the percentage of ROS-producing cells between Day 1 DMSO negative control cells and Day 1 PMA-stimulated cells (*p* = 0.013), and a significant increase between Day 5 DMSO negative control cells and Day 5 PMA-stimulated cells (*p* = 0.008). While there was an overall ~three-fold increase in the percentage of ROS-producing cells between Day 1 (2.95%) and Day 5 (9.73%) for the DMSO negative control conditions, it was not significant (*p* = 0.171). There was also no significant difference in the percentage of ROS-producing cells between Day 1 and Day 5 PMA-stimulated conditions (*p* = 0.879).

To determine whether the maximum rate of ROS production changes during culture duration, on Day 1 and Day 5, cells were treated with PMA or DMSO, and analyzed via Oroboros respirometry (Figure 1F). On Day 1, the maximum rate of ROS production was 0.09 ± 0.02 pmol/(s*million cells) in the DMSO negative control cells, and 8.90 ± 2.51 pmol/(s*million cells) in the PMA-stimulated cells. On Day 5, the maximum rate of ROS production was 0.10 ± 0.01 pmol/(s*million cells) in the DMSO negative control cells, and 7.65 ± 2.22 pmol/(s*million cells) in the PMA-stimulated cells (Figure 1F). There was a significant increase in the maximum rate of ROS production between Day 1 DMSO negative control cells and Day 1 PMA-stimulated cells (*p* = 0.039), and a significant increase between the Day 5 DMSO negative control and Day 5 PMA-stimulated cells (*p* = 0.042). There was no significant difference in the maximum rate of ROS production between Day 1 DMSO negative control and Day 5 DMSO negative control cells (*p* = 0.175) and no significant difference in the maximum rate of ROS production between Day 1 PMA-stimulated and Day 5 PMA-stimulated cells (*p* = 0.097).

To provide further confirmation that the HKLs become more macrophage-like during culture time, we examined changes in the mRNA expression of two macrophage markers, macrophage receptor with collagenous structure (*marco*) and major histocompatibility complex II (*MHC II*) [10,33,34,35,36,37,38] in the samples used for miRNA qPCR validation. There was a significant increase in the mRNA expression of both *marco* and *MHC II* in Day 5 HKLs compared to Day 1 HKLs (see Supplementary File S1 for information on this experiment).

### 2.2. Library Preparation, Deep Sequencing, miRNA Diversity Estimation and Differential Expression Analysis of Small RNA Sequence Data

The total number of reads obtained from sequencing the small RNA libraries ranged from 8.3 million to 29.3 million. Following trimming and size filtering, the percentage of reads that mapped to the Atlantic salmon miRNAome ranged from 76.6% to 95.4% (5.5 million reads to 7.2 million reads) (Table 1) [39]. All deep sequencing reads have been submitted to the NCBI Sequence Read Archive (SRA) database (see accession numbers in Table 1).

**Figure 2:**
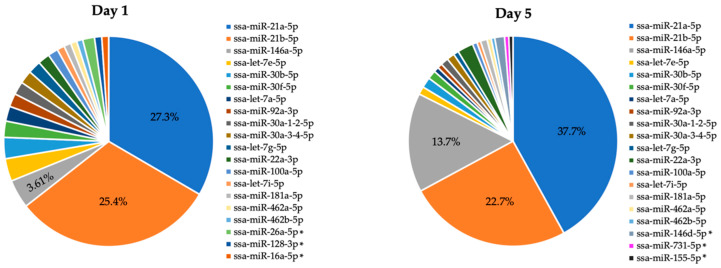
miRNA expression and diversity (average normalized read counts of 370 miRNAs) in Day 1 and Day 5 Atlantic salmon adherent HKLs. The top 20 most abundant miRNAs in Day 1 and Day 5 are indicated. * indicates miRNAs present in the top 20 of one day but not the other day.

Analysis of miRNA diversity and abundance showed that 370 out of 589 known mature Atlantic salmon miRNAs [39] are expressed in Day 1 monocyte-like HKLs and Day 5 macrophage-like HKLs. None of these were exclusively expressed in Day 1 cells or Day 5 cells. The top 20 most abundant miRNAs in both Day 1 and Day 5 HKLs are shown in Figure 2, while the abundance of all 370 miRNAs can be found in Supplementary Table S1. Three mature miRNAs—miR-21a-5p, miR-21b-5p and miR-146 a-5p—predominated in both Day 1 and Day 5 HKLs, accounting for 56.3% of all miRNAs expressed in Day 1 cells and 74.1% of all miRNAs in Day 5 cells. While miR-21a-5p and miR-146a-5p were among the highest expressed miRNAs in both days, there was an obvious increase in the proportion of both miRNAs in Day 5 cells compared to Day 1 cells. To further investigate changes in the expression of any of the identified miRNAs, we carried out differential expression analysis of the small RNA-sequenced samples from Day 1 vs. Day 5 cells.

**Figure 3:**
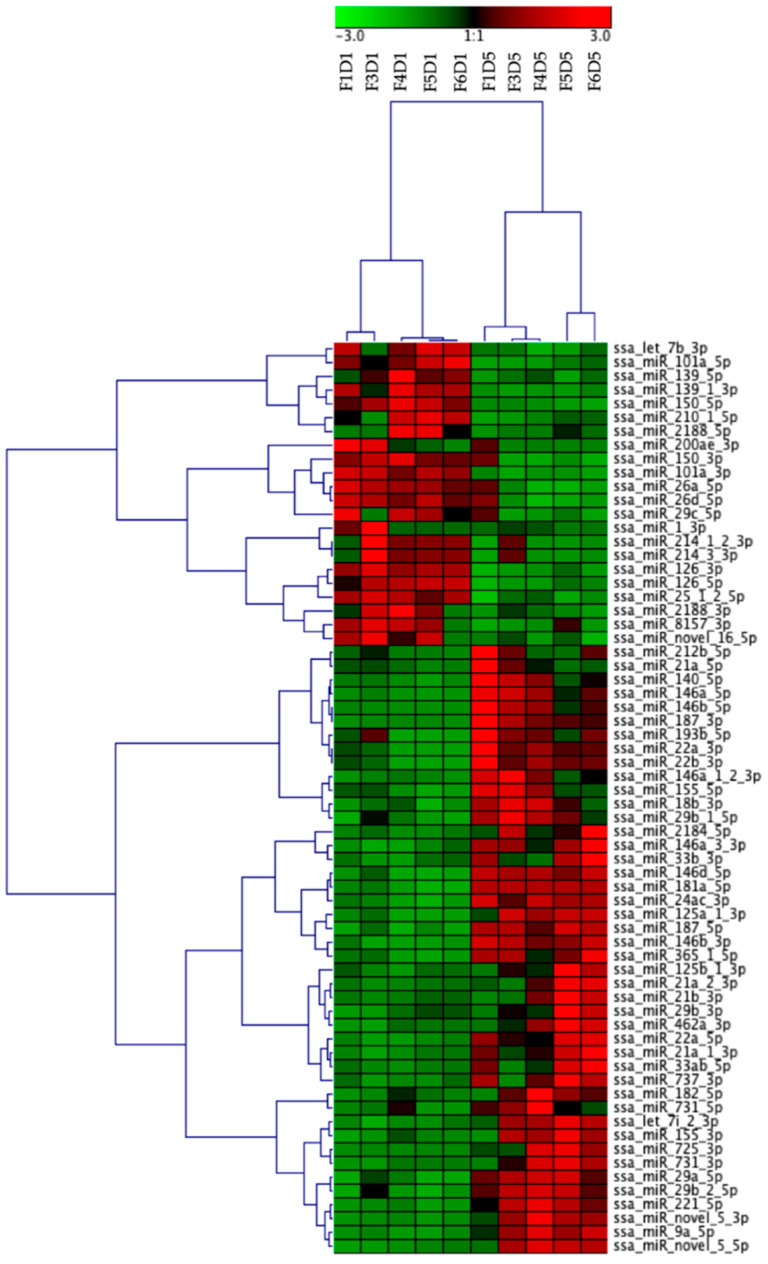
Results from hierarchical clustering of differentially expressed (DE) miRNAs from Day 1 and Day 5 HKLs shown as a heatmap. miRNAs counts per million were normalized and clustered using Pearson correlation and complete linkage hierarchical clustering. F indicates fish number; D indicates Day 1 or Day 5 (for example, F1D1 is Fish 1 Day 1).

Sixty-six miRNAs were found to be differentially expressed (DE) between Day 1 and Day 5 cells. However, a large number of these DE miRNAs were major and minor mature miRNAs from the same precursor or miRNAs from the same families. Thus, there were only 40 different miRNA families represented in the DE miRNAs. Twenty-two miRNAs from 15 miRNA families were downregulated in Day 5 HKLs compared to Day 1 HKLs while 44 miRNAs from 25 families were upregulated in Day 5 HKLs compared to Day 1 HKLs. miR-21a-5p, miR-146a-5p, miR-22a-3p, miR-181a-5p, miR-26a-5p, miR-462a-5p and miR-462b-5p were among the top 20 most abundant miRNAs that also demonstrated differential expression between Day 1 and Day 5 cells. Hierarchical clustering of the DE miRNAs showed that all Day 5 samples clustered separately from all Day 1 samples (Figure 3). Supplementary Table S2 gives a complete summary of the DESeq2 analysis results including the fold-change and mature sequence of each DE miRNA.

### 2.3. qPCR Validation of DESeq2-Identified miRNAs

**Figure 4:**
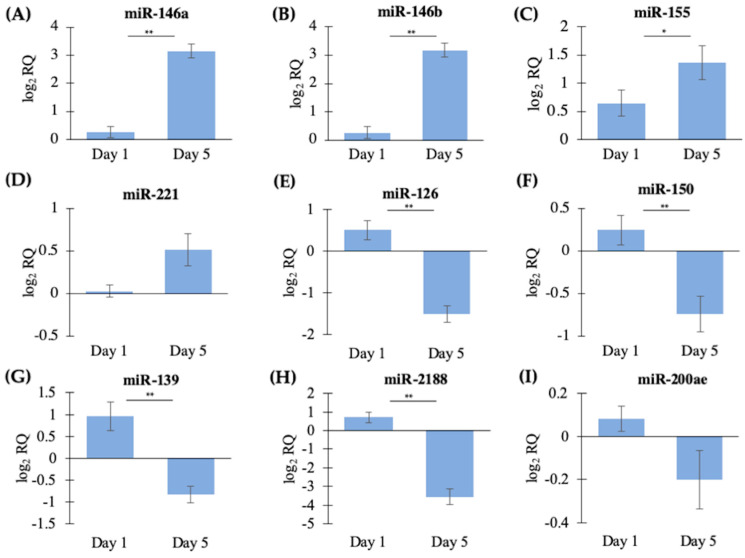
qPCR analysis of DE miRNAs identified by small RNA sequencing. Data shown as the mean log_2_ relative quantity (RQ) ± SE., *n* = 5. Significance determined by a Student’s *T*-test, * *p* < 0.05 and ** *p* < 0.01. (**A**) miR-146a, (**B**) miR-146b, (**C**) miR-155, (**D**) miR-221, (**E**) miR-126, (**F**) miR-150, (**G**) miR-139, (**H**) miR-2188 and (**I**) miR-200ae.

Nine DE miRNAs that were the major expressed mature miRNA in their families were chosen for qPCR validation. The chosen miRNAs were a combination of upregulated and downregulated miRNAs in the DESeq2 analysis and are known immune- or macrophage-related miRNAs in mammalian and/or fish literature [16,17,20,40,41]. Three miRNAs were validated as significantly upregulated in Day 5 cells compared to Day 1 cells (Figure 4A–C), while four miRNAs were validated as significantly downregulated in Day 5 cells compared to Day 1 cells (Figure 4E–H). The expression levels of two miRNAs (miR-221, *p* = 0.67; miR-200ae, *p* = 0.06) were not found to be significantly different by qPCR analysis. However, they followed the same trend as the sequencing results (Figure 4D,I). Table 2 shows a comparison of the sequencing and the qPCR results of the significantly DE miRNAs. In summary, the qPCR experiment (which analyzed HKL RNA from a different set of Atlantic salmon than the sequencing experiment) supported the differential expression analysis findings of the small RNA sequencing study.

### 2.4. In Silico Target Gene Predictions

The nine qPCR-validated miRNAs, representing the major expressed mature miRNAs from their families, were used as input for target gene predication by in silico analysis against the 3′UTRs from all Atlantic salmon transcripts in the NCBI Reference Sequence database (https://www.ncbi.nlm.nih.gov/refseq). A total of 771 genes were predicted as putative targets of one or more of the DE miRNAs (Supplementary Table S3). Predicted targets selected for discussion can be found in Table 3. miR-126-3p had the lowest number of predicted targets (4), while miR-2188-3p had the highest number of predicted targets (239). Many of the potential targets are known to play a role in macrophage differentiation and/or function in other species. For example, transcripts encoding transcription factors (e.g., SREBPs and IRF5) and cytokines (e.g., TNF-α) were identified as predicted targets of one or more of the DE miRNAs (see Section 3.3 for a more thorough discussion on the potential targets).

## 3. Discussion

Aquaculture production in Canada has increased four-fold since the early 1990s. Atlantic salmon is the top aquaculture export in Canada and is of significant economic value. Therefore, elucidating how their immune cells develop and function is a key step toward better understanding their immune system and, therefore, improving our ability to maintain healthy farmed salmon. The current study identified a change in the morphology of Atlantic salmon adherent HKLs from Day 1 (predominantly rounded, i.e., monocyte-like) to Day 5 (predominantly spread with pseudopodia present, i.e., macrophage-like) in culture, suggesting that the cells are differentiating over time. In addition to analyzing the morphology of Day 1 and Day 5 HKLs, we also analyzed phagocytosis and ROS production, both functions of macrophages. There was a higher percentage of phagocytic cells in the Day 5 culture compared to the Day 1 culture, supporting the hypothesis that these cells are differentiating into macrophages during culture time. A change in morphology and phagocytic ability has also been observed in common carp (*Cyprinus carpio* L.) and goldfish HKLs during culture duration [26,42]. However, there was no change in ROS production between Day 1 and Day 5 HKLs. It is important to note that PMA is used to differentiate monocytes to macrophages in several mammalian models [43,44,45] and is a strong inducer of ROS production. Thus, it is possible that using such a strong inducer may have masked any differences in ROS production between the two cell populations. Finally, there was a significant increase in the mRNA expression of two macrophage markers, *marco* and *MHC II*, in Day 5 HKLs compared to Day 1 HKLs (Supplementary File S1).

Studies in other species have shown that miRNAs are important regulators of macrophage differentiation and polarization [46]. We hypothesized that Atlantic salmon monocyte-like and macrophage-like HKLs would have differences in their miRNA complement and expression which likely have important roles in macrophage differentiation; we used RNA-seq and qPCR-based miRNA identification and expression analyses of cultured salmon HK monocytes/macrophages to test this hypothesis. We sought to validate the sequencing results by using a different miRNA detection method (qPCR) and new sample material. In general, there was good agreement between the smaller set of nine miRNAs analyzed by qPCR and the sequencing for those miRNAs, which provided support for the sequencing results and DESeq2 analysis. Identifying changes in miRNA expression throughout culture time will help reveal what miRNAs may have important functions in these cells, as well as what miRNAs are likely involved in regulating their differentiation and function.

### 3.1. miRNA Diversity and Abundance in Atlantic Salmon Adherent HKLs

miRNA diversity and abundance in Atlantic salmon adherent HKLs have never been reported. MicroRNAs with high abundance and ubiquitous expression in a cell population may play a role in function and in maintaining important lineage-specific gene regulation. This study identified 370 miRNAs in Atlantic salmon adherent HKLs (Figure 2 and Supplementary Table S1). Two miRNAs of the miR-21 family (miR-21a-5p and miR-21b-5p) were found to be the top two most abundant miRNAs in both Day 1 (27.3% and 25.4%) and Day 5 (37.7% and 22.7%) HKLs, while miR-146a-5p was the third most abundant miRNA in both Day 1 (3.61%) and Day 5 (13.7%) cells. Together, these miRNAs compose over half of all miRNAs in both Day 1 and Day 5 cells, suggesting that they have important functions in Atlantic salmon monocytes and macrophages.

A study by Woldemariam et al. (2019) identified the top 10 most abundant miRNAs across several tissues in Atlantic salmon, which accounted for more than 30% of the miRNAs expressed in any tissue [39]. Two of the top 20 most abundant miRNAs identified in this current study (miR-21b-5p and miR-26a-5p) were among the top 10 most abundant miRNAs in the ten tissues investigated, suggesting that these miRNAs are constitutively highly expressed and may have housekeeping functions. In addition, miR-21b-5p, miR-21a-5p and miR-146a-5p were among the most abundant miRNAs in HK tissue [39]. Interestingly, the proportion of these miRNAs was more pronounced in the isolated adherent HK cells of this current study, compared to the HK tissue in Woldemariam et al. (2019) [39]. This is likely due to the higher number of different cell types within the HK organ, compared to the isolated and cultured HKLs. The miR-21 family was also found to be among the most abundant miRNAs in the HK and spleen of Atlantic salmon that were infected with *Piscirickettsia salmonis*, as well as the control group that was not infected [47].

In other species, miR-21 is involved in macrophage differentiation, immune response and polarization, suggesting that this miRNA may have more than just housekeeping roles in leukocytes. For example, in both humans and mice, miR-21 promotes the differentiation of granulocyte-macrophage progenitors (GMP) into monocytes [46,48]. Barnett et al. (2016) found that the overexpression of miR-21 in mouse LPS-treated peritoneal macrophages decreased the expression of the inflammatory cytokine TNF-α, while suppression of miR-21 increased TNF-α and IL-6 [49]. Wang et al. (2015) found that miR-21 impaired the expression of the M2 macrophage markers arginase and resistin-like molecule alpha (*Retnla*) while enhancing the expression of the M1 markers TNF-α and IL-1β [50]. Interestingly, there is an IRF8-transcription factor binding motif upstream of all of the Atlantic salmon miRNA-21 family genes [40]. This transcription factor is known to induce transcription of genes important in murine monocyte-to-macrophage differentiation [51]. Similarly, miR-146a, the third most abundant miRNA in this study, is also involved in macrophage differentiation and function in other vertebrates [17,52,53,54,55] (see Section 3.2 for discussion on miR-146a).

### 3.2. Expression Analysis Identified DE miRNAs Known to Be Involved in Macrophage Function or Differentiation in Other Species and/or Immune Function in Atlantic Salmon

In addition to characterizing the miRNAs in Day 1 and Day 5 adherent HKLs, this study identified and confirmed miRNAs that are differentially expressed in these cell populations. MicroRNAs that are differentially expressed between Day 1 cells (monocyte-like cells) and Day 5 cells (macrophage-like) are likely involved in HK monocyte-to-macrophage differentiation and/or function via gene regulation. Many of the miRNAs identified in this study are known to be involved in macrophage function and/or differentiation in other species. In addition, several miRNAs only identified in Atlantic salmon [39] were also identified as differentially expressed between Day 1 and Day 5 cells.

With respect to miR-146a-5p, along with being the third most abundant miRNA in both Day 1 and Day 5 cells, there was a large increase in the proportion of miR-146a-5p in Day 5 cells (13.7%) compared to Day 1 cells (3.6%). The increase in the expression of miR-146a-5p in Day 5 cells was identified by both the miRNA-seq and the qPCR analyses. miR-146a and miR-146b (also differentially expressed in this study) are involved in macrophage differentiation, polarization and inflammatory response in other species. For example, the ectopic expression of miR-146a can drive maturation of human hematopoietic stem cells (HSCs) to peritoneal macrophages during adult hematopoiesis in vivo, while knockdown of miR-146a diminished macrophage formation in early zebrafish (*Danio rerio*) embryos [52]. In addition, PU.1, a transcription factor that favors differentiation of GMPs to the monocyte lineage, induces the expression of a subset of miRNAs, including miR-146a. miR146a may also have a role in macrophage polarization; downregulation of miR-146a was found in M1-polarized murine bone marrow-derived macrophages (BMDMs) compared to M2-polarized macrophages, while in human peripheral blood mononuclear cells (PBMCs), miR146a was upregulated in M1 macrophages compared to M2 macrophages [18,41]. miR-146b was found to be upregulated in M2c human PBMCs [41]. In addition, both miR-146a and miR-146b have been identified as inflammation-sensitive miRNAs as both are LPS responsive [54]. Inhibition of miR-146a upregulates M1-associated genes in LPS-stimulated RAW264.7 macrophages and decreases M2-associated genes, while the overexpression of miR-146a increases the expression of anti-inflammatory IL-10 and arginase-1, markers of M2 macrophages [55]. In several fish species, miR-146 expression changes in various tissues in response to bacterial and viral challenge. For example, both miR-146a and miR-146b expression increased in cardiac tissue of Atlantic salmon following salmonid alphavirus (SAV) infection [40]. Similarly, miR-146a expression increased in the head kidney of Atlantic salmon intraperitoneally injected with poly(I:C) or formalin-killed *Aeromonas salmonicida* compared to a saline-injected control group [56]. In grouper macrophages, miR-146a was significantly upregulated following infection with Singapore grouper iridovirus (SGIV), while inhibition of miR146a in these macrophages had an anti-viral effect, decreasing SGIV replication [24]. The results presented here show an increase in the proportion of miR-146a and miR-146b in Day 5 macrophage-like cells compared to Day 1 monocyte-like cells, suggesting that these cells are differentiating (or have differentiated) toward the macrophage lineage. In addition, the data presented here also suggest that miR-146a and miR-146b may have similar functions in Atlantic salmon as other species; however, functional studies, as well as specific M1/M2 gene expression studies, are required to test this hypothesis.

miR-155 is also involved in macrophage differentiation and function in other species. For example, miR-155 is, like miR-146a and miR-146b, induced by PU.1, suggesting that it has a role in myeloid cell differentiation [52]. In addition, a study by Mann et al. (2010) demonstrated that commitment of the mouse monocyte cell line RAW264.7 to active macrophages involves the upregulation of miR-155 expression [57]. Infection of mammalian macrophages with *Listeria monocytogenes* or *Mycobacterium avium* upregulated miR-155 expression, as well as miR-146a and miR-146b expression, suggesting their involvement in regulating the response to bacterial infections [58,59]. Likewise, miR-155 expression increased in response to both LPS and poly(I:C) [54,60]. An in vitro study in the teleost fish species ayu (*Plecoglossus altivelis*) found that infection of ayu macrophages with *Vibrio anguillarum* increased the levels of miR-155. Furthermore, the overexpression of miR-155 in the *V. anguillarum*-infected macrophages enhanced the expression of pro-inflammatory cytokines (IL-1β, TNF-α) and decreased the expression of anti-inflammatory cytokines (IL-10, TGF-β) compared to the control, while inhibition of miR155 had the opposite effect [22]. In addition, miR-155 promoted M1-type polarization and inhibited M2-type polarization, suggesting that, similar to higher vertebrates, miR-155 may play a role in macrophage function and polarization in this fish species [22].

There is little known regarding the involvement of miR-150 and miR-126 in monocyte-to-macrophage differentiation and function. In mammals, miR-150 plays a central role in B cell development, where it is expressed in mature lymphocytes but not their progenitors [61]. miR-150 was identified as differentially regulated during the maturation of human monocytes into macrophages, where it was downregulated in macrophages compared to monocytes [41]. miR-150 expression was also decreased in LPS-stimulated murine BMDMs, which negatively correlated with PU.1 transcript expression, suggesting that miR-150 is inflammation responsive and may also interact with PU.1 [62]. Luciferase assays confirmed that miR-150 directly targeted the PU.1 transcript. Furthermore, the overexpression of miR-150 in murine BMDMs significantly reduced PU.1 transcript expression and shifted polarization away from M1 [62]. These results suggest that miR-150 can alter macrophage activation and inflammatory response. The role and function of miR-150 in teleost macrophages remain to be determined. We found a decrease in miR-150 expression in Day 5 macrophage-like HKLs compared to Day 1 monocyte-like cells, suggesting that if miR-150 has the same role in Atlantic salmon as it does in other vertebrates, then both the expression changes and the morphology analysis indicate that the HKLs become more macrophage-like and less monocyte-like during culture time. miR-126, along with a subset of miRNAs, is enriched in human and murine HSCs, compared to other cells in the bone marrow, and its expression is decreased in differentiated cells of the lymphoid and myeloid lineages, as determined by a bidirectional miR-126 reporter vector [63]. Likewise, knockdown of miR-126 increased mouse and human HSC proliferation, while the overexpression of miR-126 increased HSC quiescence [64]. In fish, miR-126 is involved with vascular, oocyte and early embryo development. However, it is unknown whether it plays a role in monocyte-to-macrophage differentiation and/or function [65,66,67,68]. The results of the current study found a higher expression of miR-126 in Day 1 cells compared to Day 5 cells, suggesting that, like miR-150, if miR-126 plays a similar role in Atlantic salmon as it does it mammals, then the Day 1 HKL population consists mostly of early, undifferentiated myeloid cells, such as monocytes, while the Day 5 HKL population consists mostly of differentiated myeloid cells, such as macrophages. The role of both miR-150 and miR-126 in Atlantic salmon monocyte-to-macrophage differentiation and function warrants further investigation.

miRNA-2188, miRNA-462 and miR-731 are teleost-specific miRNAs that have not yet been discovered in mammals and are associated with immune responses in fish including Atlantic salmon [20,40]. In the olive flounder and in the Atlantic salmon, miR-2188 expression decreased in the head kidney following viral hemorrhagic septicemia infection and in cardiac tissue following salmonid alphavirus infection, respectively [40,69]. Conversely, miR-2188 expression increased in Atlantic cod macrophages in response to poly(I:C) stimulation at 48 h and 72 h post-stimulation [23]. Interestingly, miR-2188 was significantly downregulated in unstimulated cod macrophages at 72 h compared to cells cultured for 12 and 24 h [23]. Similar to the cod macrophages, we saw a decrease in miR-2188 expression after 5 days of culture. Therefore, it is possible that miR-2188 plays a conserved role in teleost fish monocyte-like and macrophage-like cells. DESeq2 analysis of miRNA-seq data herein showed that three novel Atlantic salmon miRNAs [39] were differentially expressed between Day 5 and Day 1 cells (e.g., miR-novel-5-3p and miR-novel-5-5p were upregulated in Day 5 cells; miR-novel-16-5p was downregulated in Day 5 cells). Novel miRNAs may be species specific, as they are absent or have not been identified in other species including higher vertebrates; this suggests that some aspects of miRNA roles in Atlantic salmon macrophage differentiation may be species specific.

### 3.3. In Silico Target Prediction Identified Potential Targets Involved in Macrophage Differentiation and Function in Other Species

The potential target genes of the nine qPCR-validated DE miRNAs were identified as they represent the major expressed mature miRNAs from eight of the 40 different families. Applying such predictions on these validated DE miRNAs could reveal whether they could target genes relevant to macrophage function and differentiation. A total of 771 genes were predicted as putative targets for one or more of the DE miRNAs, with a range of 4 to 239 target genes per miRNA (Supplementary Table S3). Several of the targets are known to be involved in macrophage activation, immune response and cell differentiation. In mammals, the lipidomic and transcriptomic profiles change profoundly during macrophage differentiation and lipid metabolism plays a key role in macrophage activation and function [70,71]. Potential targets of the DE miRNAs identified and validated in this study include various lipid-related transcripts such as sterol regulatory element-binding proteins 1 and 2 (SREBP1 and SREBP2 (also known as SREBF1 and SREBF2); potential targets of miR-139-5p and miR-200ae-3p), and elongation of very-long-chain fatty acids protein 5 and 7 (ELOVL5, ELOVL7; potential targets of miR-139-5p, miR-155-5p, miR-200ae-3p and miR-221-5p). SREBPs are key transcription factors in the synthesis of fatty acids and cholesterol. An increased expression of *srebp-1a* following LPS simulation was demonstrated in mouse macrophages, while macrophages from mice with a SREBP-1a deficiency were unable to induce lipid biosynthesis in response to LPS. They also displayed a decreased level of Il-1β cytokine secretion [72]. In addition, Ecker et al. (2010) demonstrated that a SREBP1-dependent induction of human monocyte fatty acid synthesis is vital for monocyte-to-macrophage differentiation, while Lee et al. (2018) determined that phagocytosis is impaired in cells that lack a key SREBP isoform [73,74]. In monocyte-to-macrophage differentiation of a human cell line, *elovl5* mRNA expression was strongly induced, while fatty acyl-CoA reductase (a potential target of miR-221-5p) mRNA expression was downregulated [70].

Several transcription factors with known functions in macrophage M1/M2 polarization were identified as potential miRNA targets. Interferon regulatory factor 5 (IRF5) was identified as a putative target for miR-200ae-3p. In mammalian macrophages, IRF5 is a regulator of M1 macrophage polarization; M1 macrophages have higher mRNA and protein expression of IRF5 compared to M2 macrophages [75,76]. The forced expression of IRF5 in human M2 macrophages strongly induced the mRNA expression of M1-specific cytokines, while reducing the mRNA expression of the anti-inflammatory cytokine IL-10. Conversely, knockdown of IRF5 in M1 macrophages inhibited the LPS-induced expression of pro-inflammatory cytokines [75]. In common carp, *irf5* mRNA expression increased in several immune tissues, including the HK, following poly(I:C) challenge, suggesting that it plays a role in regulating immune response in fish [77]. C/EBPα (a potential target of miR-2188-3p) and C/EBPβ (a potential target of miR-200ae-ep) are also important transcription factors in regulating polarization of M1/M2 macrophages [78,79]. Macrophages from C/EBPα-deficient mice exhibited a decreased expression of M1 and M2 markers following LPS and IL-4 stimulation, respectively, suggesting that C/EBPα plays a role in both M1 and M2 polarization [80]. Similarly, in LPS/IFN-γ-stimulated mouse macrophages, impaired C/EBPβ expression was associated with the suppression of M2 markers, while M1 markers were unaffected [81]. In mammalian macrophages, C/EBPβ is a direct target of miR-155 [82]. GATA3, which was identified as a target of both miR-146a-5p and mir-146b-5p in the current study, has been associated with M2 polarization in mouse macrophages [83]. Treatment of mouse monocytes with *gata3* shRNA decreased the expression of M2 markers (Arg1, IL-4), while the forced expression of *gata3* downregulated the expression of M1 markers (TNF-α, MCP-1, CD206) but induced the expression of M2 markers (Arg1, IL-1, iNOS) [83].

Transcripts encoding several cytokines, chemokines and other inflammatory and macrophage-related proteins were also identified as potential miRNA targets in the current study. For example, the transcript encoding tumor necrosis factor receptor superfamily member 1A (TNFRSF1A) was identified as a potential target of miR-139-5p, miR-155-5p and miR-2188-3p. TNFRSF1A is one of the major receptors for TNF-α (a potential target of miR139-5p) which is produced primarily by monocytes and macrophages and plays a role in critical cell processes including inflammation and differentiation [84,85,86]. In fish, in vitro treatment of primary rainbow trout (*Oncorhynchus mykiss*) HKLs with TNF-α induced the expression of several inflammatory genes, including *il1b*, *il8, il17c, tnfa* and *cox2* and enhanced phagocytic activity [87]. TNF-α also plays a role in macrophage differentiation. An increase in *tnfa* gene expression is observed during differentiation of BMDMs [88]. Blocking the increase in *tnfa* expression by using anti-sense oligomers prevented macrophage differentiation, causing the cells to proliferate instead [88]. In the current study, the transcript encoding the protease legumain was identified as a potential target of miR-150-5p. Legumain expression and secretion are increased during human monocyte-to-macrophage differentiation, with M2 macrophages expressing significantly higher mRNA levels and secretion of legumain than M1 macrophages [89]. In goldfish HKLs, the mRNA expression of legumain was highest in mature macrophages, compared to early progenitor and monocyte populations [90]. In this study, miR-150 expression was lower in Day 5 cells compared to Day 1 cells, which would be expected if legumain transcript is a target of miR-150. Also in goldfish HKLs, granulin transcript, a potential target of miR-155-5p in the current study, is more highly expressed in monocytes compared to early progenitor cells and mature macrophages [90]. Macrophages isolated from CCR6 (a potential target of miR-200ae-3p) deficient mice had lower levels of inflammatory cytokines following LPS stimulation, compared to WT macrophages [91]. In addition, there was a significantly higher mRNA expression of CCR6 in human M1 macrophages than in M2 macrophages, while CCL25 (a target of miR-155-5p) induced chemotaxis of M1 macrophages [92].

Taken together, the results of this in silico target prediction analysis with the qPCR-validated miRNAs suggest that many of the DE miRNAs in this study may target genes that are involved in macrophage differentiation, function and immune response, similar to other species. It is important to note that there are often many false positives identified during target prediction. This is in part due to the lack of consistency in target prediction tools, and that the rules for governing miRNA target recognition are not fully understood, and so can vary for each miRNA target prediction [20,93,94]. In addition, we only examined target miRNAs from a small subset of DE miRNAs. Validation studies, including overexpression and/or knockdown studies, are required to confirm that a miRNA–target interaction is authentic.

## 4. Materials and Methods

### 4.1. Animals

Atlantic salmon were reared in the Dr. Joe Brown Aquatic Research Building (JBARB) of the Ocean Sciences Centre in 3800 L tanks and kept at 12 °C with 95–110% oxygen saturation, using a flow-through seawater system. All procedures in this experiment were approved by Memorial University of Newfoundland’s Institutional Animal Care Committee (protocol 18-01-MR, approved 15 May 2018) based on the guidelines of the Canadian Council of Animal Care. A total of 19 salmon (1.8 kg +/− 0.5 kg) were used in this study as follows: 4 individuals were used for morphology analysis and phagocytosis assays (Section 4.3 and Section 4.4), 4 individuals were used for both respiratory burst assays (Section 4.5), 6 individuals were used for miRNA sequencing with 1 individual excluded from analysis (see Section 4.6 for explanation), and 5 individuals were used for qPCR validation of miRNA results (Section 4.9). Three of the 5 individuals used for qPCR validation of miRNA sequencing results were randomly selected and used to examine *marco* and *MHC II* mRNA expression (methods in Supplementary File S1).

### 4.2. Adherent HKL Isolation and Culture

Adherent HKLs were isolated as previously described, with some modifications [30,95]. Briefly, the head kidney was removed and placed in isolation media: 500 mL of Leibovitz-15 medium (l-15 Gibco, Carlsbad, CA, USA) supplemented with 2.5% fetal bovine serum (FBS, Gibco), 1% penicillin/streptomycin (Gibco) and 27.5 mg of heparin (Sigma-Aldrich, St. Louis, MO, USA). The head kidney cells were forced through a 100 µM nylon cell strainer (Thermo-Fisher Scientific, Waltham, MA, USA), placed on a 34/51% Percoll (GE Healthcare, Uppsala, Sweden) gradient (prepared with H_2_O and 10X Hank’s Balanced Salt Solution (HBSS; Sigma-Aldrich) to ensure an isotonic solution), and centrifuged at 500× *g* for 30 min at 4 °C. Following centrifugation, the interface between the 34% and 51% gradient, which contains leukocytes, was collected and washed twice in isolation media at 500× *g* for 5 min at 4 °C. The cells were re-suspended in culture media (L-15 supplemented with 5% FBS and 1% penicillin/streptomycin), and viable cells were counted on a hemocytometer using Trypan Blue (Sigma-Aldrich) exclusion. The cells were then seeded in 6-well culture plates (Corning, Corning, NY, USA) at 1 × 10^7^ cells (for Giemsa staining) or 3 × 10^7^ cells (for RNA extraction) in 2 mL of culture media per well and incubated at 15 °C for 24 h to allow cell adherence. Following the 24 h incubation, cells were washed twice in culture media to remove non-adherent cells, and the media was replaced with fresh culture media. Media was changed every 48 h thereafter. The cells were cultured for up to 5 days.

### 4.3. Morphology Analysis

Twenty-four hours (Day 1) and 120 h (Day 5) after seeding, cells were washed twice with PBS then 1.0 mL of Giemsa stain (Thermo Fisher Scientific) was added directly to the culture plate for 3 min. The Giemsa stain was then removed and replaced with 2.0 mL of PBS for 6 min. The cells were then rinsed with PBS until the edges of the well were slightly pink and excess stain was removed. The cells were then air dried and images were taken on an Eclipse Ti-S inverted microscope immediately following air drying. The morphology of the cells was analyzed by counting approximately 200 cells from at least 3 fields of view from each fish and defining the cells as either round (non-spread; no pseudopodia visible) or spread (cells with pseudopodia present). A chi-square test was performed to determine whether changes in the proportion of round and spread cells in Day 1 vs. the proportion of round and spread cells in Day 5 were significant using GraphPad Prism v 8.0 (GraphPad Software Inc., La Jolla, CA, USA).

### 4.4. Phagocytosis Assay

Twenty-four hours (Day 1), 72 h (Day 3) and 120 h (Day 5) after seeding, cells were washed twice in culture media, and 1 µm Fluoresbrite YG microspheres (Polysciences, Warrington, PA, USA) were added at a ratio of approximately 1:30 macrophage:microsphere [29,96]. Twenty-four hours after microsphere addition, the cells were washed twice with culture media, followed by removal from the plate using trypsin-EDTA (0.25%) (Thermo Fisher Scientific) and then re-suspended in 500 µL of fluorescence-activated cell sorting (FACS) buffer (phosphate-buffered saline (PBS) + 1% FBS). Fluorescence was detected from 10,000 cells using a BD FACS Aria II flow cytometer and analyzed using BD FACS Diva v7.0 software (BD Biosciences, San Jose, CA, USA). FITC-positive cells, based on gating using the negative/unstained cells, were identified and the percentage of FITC-positive cells was determined. A one-way ANOVA was performed to determine significant differences between the percentage of phagocytic cells between Day 1, Day 3 and Day 5 cells using GraphPad Prism v 8.0.

### 4.5. Respiratory Burst Assays

A change in morphology between Day 1 and Day 5 cells was confirmed visually before sampling. To examine the respiratory burst response using flow cytometry, on Day 1 and Day 5 of culture, the culture media was removed and replaced with 500 µL of respiratory burst assay buffer (L-15 media + 1% BSA + 1 mM CaCl2). One microliter of dihydrorhodamine 123 (DHR; Sigma-Aldrich) (5 mg/mL) was diluted in 1 mL of PBS and 50 μL of the dilution was added to the cells for 15 min. Following DHR addition, 1 μL of 1 mM phorbol myristate acetate (PMA; Sigma-Aldrich) dissolved in dimethyl sulfoxide (DMSO; Sigma-Aldrich) was diluted in 1 mL of respiratory burst assay buffer and 125 μL of this solution (or an equal volume of respiratory burst assay buffer containing 0.1% DMSO for a negative control) was added to the cells (i.e., final concentration of 0.185 μM PMA) for 45 min to stimulate ROS production [97]. Cells were removed from the plate using trypsin-EDTA (0.25%), centrifuged for 5 min, 500× *g*, at 4 °C and re-suspended in FACS buffer. Fluorescence was detected from 10,000 cells using a BD FACS Aria II flow cytometer and analyzed using BD FACS Diva v7.0 software. The DMSO vehicle control cells were used to define the region of ROS-negative cells and based on this gating, the FITC-positive cells were identified.

To examine the respiratory burst response using the Oroboros (Oroboros Instruments, Innsbruck, Austria), on Day 1 and Day 5, cells were removed from the plate using trypsin-EDTA (0.25%), centrifuged for 5 min, 500× *g*, at 4 °C, counted and 2 × 10^6^ cells were re-suspended in 50 μL of L-15 + FBS. Cells were placed in the 2 mL Oroboros chamber containing L-15 + 1% FBS at 15 °C for 20–30 min to equilibrate. The rate of ROS production was estimated by measuring extramitochondrial H_2_O_2_ detected by the green fluorescence sensor of the O2k-Fluo LED2 module (with gain and LED intensity set to 1000 and 500 mV, respectively) with Amplex^®^ UltraRed (10 µmol L^−1^, Thermo Fisher Scientific), horseradish peroxidase (3 U mL^−1^, Sigma-Aldrich), and SOD (U mL^−1^, Sigma-Aldrich). The ROS signal was calibrated by the addition of H_2_O_2_ (0.1 μmol L^−1^) before and after adding cells to the chamber. After 20–30 min, ROS was stimulated by adding 4 µL of 0.1 mM PMA (or the equivalent amount of DMSO for a negative control) to the chamber and the rate of ROS production was recorded in real time using DatLab 7 software (Oroboros Instruments) to obtain the maximum rate (3–15 min) of ROS production in each sample.

Paired Student’s *T*-test was used to determine statistical differences between Day 1 and Day 5 control DMSO samples, between Day 1 and Day 5 PMA samples and between control DMSO and PMA samples in each day using GraphPad Prism v 8.0.

### 4.6. Total RNA Extraction for Sequencing

A change in morphology between adherent HKLs on Day 1 and Day 5 in culture was confirmed visually before sampling. Total RNAs were extracted using the mirVana miRNA isolation kit (Thermo Fisher Scientific) according to the manufacturer’s instructions. All RNAs had a 260/280 ratio and a 260/230 ratio greater than 1.8, as determined by NanoDrop spectrophotometry, and tight 18S and 28S ribosomal RNA bands, as determined by a 1% agarose gel electrophoresis. The RNA concentration from Fish 2 was too low to proceed with RNA sequencing and therefore it was excluded from sequencing.

### 4.7. Library Preparation and Sequencing

Library construction and sequencing analyses were carried out at the Norwegian High-Throughput Sequencing Centre (NSC; Oslo, Norway). The Illumina NEBNext Multiplex Small RNA Library Preparation Kit (New England Biolabs, Inc. Ipswich, MA, USA) was used to construct 10 libraries (five Day 1 samples and five Day 5 samples) with 1 μg total RNA input, according to the manufacturer’s instructions. RNAs isolated from the same 10 samples were ligated with 3′ and 5′ RNA adapters, followed by reverse transcription and PCR enrichment using barcoded RT primers. The cDNA products were purified using 6% polyacrylamide gels, and size selection of fragments (approximately 145–160 bp) was carried out to enrich for small RNAs. Sequencing was performed on a NextSeq 500 from Illumina (Illumina, Inc, San Diego, CA, USA), producing 75 bp single-end reads.

### 4.8. Data Processing, Differential Expression Analysis and miRNA Diversity Estimation

FASTQC software (v.0.11.5; http://www.bioinformatics.babraham.ac.uk/projects/fastqc) was used to check the raw sequence reads to ensure that the data were of good quality and size for downstream analysis. The adapter sequences were then removed (trimmed) and size filtered using the Cutadapt Python Package (v.1.13) to discard reads shorter than 18 nucleotides (nts) or longer than 25 nts (i.e., outside the size range of mature miRNAs) [98]. A second FastQC analysis was performed to assess the quality of the adapter-trimmed and size-filtered sequence reads.

The sequence reads were mapped to a reference index consisting of all known mature miRNAs in Atlantic salmon using STAR aligner software (v2.4.2b) [39,99]. The alignment files (BAM format) were further processed in R using the feature counts function from the Rsubread package to produce count matrices [100]. These count tables were used as input in the R package DESeq2 to test for the differential expression of miRNAs [101]. Differentially expressed miRNAs were identified by comparing the Day 1 group (control) to the Day 5 group (*n* = 5 from each experimental condition). miRNAs were considered to be statistically differentially expressed if they had a Benjamini–Hochberg adjusted *p*-value of ≤0.05, base mean read counts ≥20 and log_2_ fold-change of ≥1 or ≤−1.

To estimate the miRNA diversity in Day 1 and Day 5 samples, the normalized read counts from the DESeq2 analysis (370 miRNAs) were exported and processed in Microsoft Excel. MicroRNAs with an average normalized read count in Day 1 cells and Day 5 cells of less than 20 were filtered out. The remaining miRNAs were used to develop a pie chart of the miRNA diversity in Day 1 cells and Day 5 cells.

The normalized data of differentially expressed miRNAs were hierarchically clustered using the Pearson correlation and complete linkage clustering function in Genesis software (Rockville, MA, USA).

### 4.9. qPCR Analysis of miRNA Expression

A change in morphology between Day 1 and Day 5 adherent HKLs was confirmed visually before sampling. Total RNA was isolated using a mirVana miRNA isolation kit, as described in Section 4.6. cDNA was synthesized using the miScript II RT Kit (Qiagen, Hilden, Germany), as per the manufacturer’s instructions, with 400 ng of total RNA in 20 μL reactions. Each qPCR reaction was composed of 12.5 μL of 2× QuantiTect SYBR Green PCR Master Mix (Qiagen), 2.5 μL of 10× miScript Universal Primer (1 μM final concentration), 2.5 μL specific forward primer (1 μM final concentration), 5 μL RNase-free water (Thermo Fisher Scientific) and 2.5 μL of diluted cDNA template representing 5 ng of input total RNA. The sequences of the mature miRNAs of interest were used as the forward specific primer, while a universal primer, provided by the miScript SYBR Green PCR Kit (Qiagen), was used as the reverse primer. Three-fold, 5-point standard curves of pooled cDNA were used to assess the quality of all primers, with the exception of miR-155-5p and miR-146a-5p, where a 4-point standard curve was used. Primer sequences, R^2^ and amplification efficiencies can be found in Table 4.

To select the normalizers used in this study, several miRNAs that demonstrated stable expression in the RNA sequencing results were tested for stability between Day 1 and Day 5 samples using qPCR [102]. The selected normalizers were expressed stably in our qPCR study (i.e., geometric mean of normalizers’ Ct less than 0.1 cycle different for Day 1 and Day 5 groups). qPCR assays for normalizers and miRNAs of interest included a no-template control and were performed in duplicate using a ViiA7 real-time PCR system (Applied Biosystems/Thermo Fisher Scientific). The PCR program consisted of one cycle of 95 °C for 15 min, and 40 cycles of 94 °C for 15 s, 55 °C for 30 s and 70 °C for 30 s, followed by a final melting point analysis.

Excel was used to determine the relative quantity (RQ) values of each miRNA relative to a calibrator (i.e., the Day 1 sample that showed the lowest expression (highest normalized Ct value: RQ = 1.0) of a given miRNA of interest compared to other Day 1 samples) [103]. Student’s *T*-test was used to determine statistically significant differences between Day 1 and Day 5 samples using GraphPad Prism v 8.0.

### 4.10. In Silico Predictions of Target Genes

Target gene predictions were carried out using the target gene prediction software RNAhybrid version 2.2.1 [104]. The mature sequences of the nine differentially expressed miRNAs analyzed by qPCR were tested against a 3′UTR dataset for 3448 genes obtained from the Refseq database of GenBank by RNAhybrid. The following parameters were used in the analysis: helix constraint 2–8, no G:U in seed, and a minimum free energy threshold of ≤−18 kcal/mol. These parameters allowed for only target genes with perfect seed complementarity and high-stability site matches from RNA hybrids to be detected.

## 5. Conclusions

This present study identified changes in miRNA expression in Atlantic salmon adherent HKLs that were cultured for 1 day or 5 days. As these cells are often used in fish in vitro immunology research, it is important to characterize how the cells change throughout culture time. Morphology and phagocytosis analyses, as well as *marco and MHC II* mRNA expression, suggested that the adherent HKLs studied differentiated from monocyte-like to macrophage-like over 5 days in culture. Sequencing analysis identified several differentially expressed miRNAs that are associated with macrophage differentiation and function in other vertebrates, indicating that the role of these miRNAs may be similar in Atlantic salmon as in other species. This is the first study to examine potential miRNAs involved in macrophage differentiation in Atlantic salmon. Future functional studies, e.g., by manipulating the expression of certain DE miRNAs in cells cultured over 5 days, are required to further elucidate and fully understand the roles of the identified miRNAs in Atlantic salmon HK cell differentiation and function.

## Figures and Tables

**Table 1:** Sequencing results.

Sample	Total Number of Reads ^a^	Trimmed and Filtered Reads ^b^	Reads Mapped to miRNA (%) ^c^	Accession Number ^d^
Fish 1 Day 1	10,954,203	6,089,270	87.9%	SRR9710703
Fish 1 Day 5	14,469,109	7,317,802	95.4%	SRR9710704
Fish 3 Day 1 ^e^	27,215,751	5,019,027	76.6%	SRR9710705
Fish 3 Day 5	19,158,159	6,104,257	88.3%	SRR9710706
Fish 4 Day 1	29,288,867	5,521,671	77.6%	SRR9710709
Fish 4 Day 5	26,403,552	6,365,057	81.9%	SRR9710710
Fish 5 Day 1	10,711,291	6,013,861	78.8%	SRR9710711
Fish 5 Day 5	8,325,813	4,870,035	79.6%	SRR9710712
Fish 6 Day 1	10,064,941	5,282,051	80.5%	SRR9710707
Fish 6 Day 5	8,760,222	6,032,420	87.1%	SRR9710708

^a^ Total number of reads in raw fastq file. ^b^ Total number of reads after removing adaptors and filtering reads by size. ^c^ Reads mapped to reference index (known miRNAs of Atlantic salmon) [39]. ^d^ Accession number of sequencing results for each sample submitted to NCBI Sequence Read Archive (SRA) database. ^e^ See Materials and Methods (Section 4.6) for an explanation for the exclusion of Fish 2.

**Table 2:** Differentially expressed miRNAs identified by sequencing and validated by qPCR.

Sequencing				qPCR	
Mature miRNA	Base Mean ^a^	log_2_ FC ^b^	Adjusted *p*-Value ^c^	log_2_ FC ^d^	*p*-Value ^e^
miR-146a-5p	737973.84	2.91	1.02 × 10^−11^	10.99	2.00 × 10^−4^
miR-146b-5p	15429.78	4.03	3.76 × 10^−17^	12.88	1.00 × 10^−4^
miR-155-5p	38126.56	1.39	5.81 × 10^−3^	2.09	3.01 × 10^−2^
miR-126-3p	11661.74	−1.52	3.51 × 10^−6^	−2.99	2.00 × 10^−4^
miR-150-5p	3140.46	−1.52	1.28 × 10^−8^	−2.97	2.00 × 10^−4^
miR-139-5p	643.30	−1.00	1.78 × 10^−2^	−0.87	5.30 × 10^−3^
miR-2188-3p	1269.71	−1.49	8.23 × 10^−3^	−5.00	2.00 × 10^−4^

^a^ The mean of normalized read counts for all of the samples. ^b^ Log_2_-transformed fold-change (FC) (Day 5/Day 1) as determined by DESeq2 analysis. ^c^ Benjamini–Hochberg *p*-value. ^d^ Log_2_ fold-change (FC). ^e^ Student’s paired *T*-test *p*-value.

**Table 3:** Selected predicted miRNA targets.

Functional Category	miRNA	Predicted Target mRNA (Gene Symbol) ^a^	Predicted Target mRNA (Gene Name)
**Lipid related**	ssa-miR-139-5p	*srebf1*	Sterol regulatory element-binding protein 1
	ssa-miR-139-5p	*srebf2*	Sterol regulatory element-binding protein 2
	ssa-miR-139-5p	*elovl5a*	Elongation of very-long-chain fatty acids protein 5
	ssa-miR-155-5p	*elovl5a*	Elongation of very-long-chain fatty acids protein 5
	ssa-miR-200ae-3p	*elovl7*	Elongation of very-long-chain fatty acids protein 7
	ssa-miR-221-5p	*elovl5*	Elongation of very-long-chain fatty acids protein 5
	ssa-miR-221-5p	*facr1*	Fatty acyl-CoA reductase
**Transcription factors**	ssa-miR-146a-5p	*gata3*	Transcription factor GATA-3
	ssa-miR-146b-5p	*gata3*	Transcription factor GATA-3
	ssa-miR-200ae-3p	*irf5*	Interferon regulatory factor 5
	ssa-miR-200ae-3p	*cebpb*	CCAAT/enhancer-binding protein beta
	ssa-miR-2188-3p	*cebpa*	CCAAT/enhancer-binding protein alpha
**Immune/macrophage related**	ssa-miR-126-3p	*i13r2*	Interleukin-13 receptor alpha-2 chain
	ssa-miR-139-5p	*tnr1a*	Tumor necrosis factor receptor superfamily member 1A
	ssa-miR-150-5p	*lgmn*	Legumain
	ssa-miR-155-5p	*tnr1a*	Tumor necrosis factor receptor superfamily member 1A
	ssa-miR-155-5p	*grn*	Granulin
	ssa-miR-155-5p	*ccl25*	C-C motif chemokine 25
	ssa-miR-200ae-3p	*ccr6*	C-C chemokine receptor type 6
	ssa-miR-2188-3p	*tnr1a*	Tumor necrosis factor receptor superfamily member 1A

^a^ Full details found in Supplementary Table S3.

**Table 4:** qPCR Primers.

miRNA	Primer Sequence 5′ to 3′ ^a^	R^2^	Amplification Efficiency (%)
miR-155-5p ^b^	TTAATGCTAATCGTGATAGGGGT	0.999	81.3
miR-146b-5p	TGAGAACTGAAGTCCATAGATGG	0.986	104.6
miR-146a-5p ^b^	TGAGAACTGAATTCCATAGATGG	0.989	115.9
miR-126-3p	TCGTACCGTGAGTAATAATGCA	0.984	107.2
miR-150-5p	TCTCCCAATCCTTGTACCAGTG	0.992	113.9
miR-2188-3p	GCTGTGTGAGGTCAGACCTATC	0.982	116.5
miR-139-5p	TCTACAGTGCATGTGTCTCCAGT	0.974	100.9
miR-221-5p	ACCTAGCATACAATGTAGATTTC	0.984	115.4
miR-200ae-3p	TAATACTGCCTGGTAATGATGAT	0.952	82.3
**Normalizers**			
miR-125a-5p	TCCCTGAGACCCTAACTTGTGA	0.994	115.1
miR-19c-3p	TGTGCAAATCCATGCAAAACTG	0.990	104.1

^a^ Mature miRNA sequences were used as the forward specific primer, whereas a universal primer was used as a reverse primer. All primers showed no amplification in the no-template controls and generated an amplicon with a single melting peak. ^b^ 4-point serial dilution curve was used.

## Supplementary Materials

Supplementary Materials can be found at https://www.mdpi.com/1422-0067/21/11/3989/s1.
