# Supplementary material for: Characterization of miRNAs in Cultured Atlantic Salmon Head Kidney Monocyte-Like and Macrophage-Like Cells
Source: Int J Mol Sci. 2020 Jun 2;21(11):3989. doi: 10.3390/ijms21113989 (PMC7312525; doi:10.3390/ijms21113989)
Supplement: Supplementary file 1 [file ijms-21-03989-s001.zip › Smith_revision_Supplemental_File_1_May25_2020_240pm_submitted2.pdf]

Supplementary File 1

Materials and Methods

mRNA expression of two macrophage markers, macrophage receptor with collagenous structure (*marco*) and major histocompatibility complex class II alpha chain *(MHC II),* was examined by qPCR in 6 HKL samples (from 3 out of the 5 fish, randomly selected) used for miRNA qPCR validation. Elongation factor 1 alpha-2 (*ef12a*) was used as a normalizer gene for this qPCR experiment. The selected normalizer was expressed stably in our qPCR study (i.e. the average Ct value less than 0.3 cycle different for Day 1 and Day 5 groups). Primer efficiencies were determined by a 5-point standard curve using pooled cDNA, starting at 10 ng of input RNA in a ViiA 7 Real-Time PCR System (Applied Biosystems). Standard curves were completed using Day 1 and Day 5 HKL samples from a different set of 5 Atlantic salmon. Reactions were run in triplicate, and no-template controls were run for each primer pair. Primer sequences and amplification efficiencies are shown in Supplementary File 1 Table.

For each qPCR reaction, Power SYBR Green Master Mix (Thermo Fisher Scientific) was mixed with 50 nM of both the forward and reverse primers and cDNA template representing 5 ng of input RNA in a ViiA 7 Real-Time PCR System. The real-time analysis program consisted of 1 cycle of 50°C for 2 min, 1 cycle of 95°C for 10 min, and 40 cycles of 95°C for 15 sec and 60°C for 1 min, with fluorescence detection at the end of each 60°C step. Reactions were run in duplicate. Each melt curve analysis showed a single amplicon peak with no primer dimers. Excel was used to determine the relative quantity (RQ) values of each mRNA relative to a calibrator (i.e. the Day 1 sample that showed the lowest expression (highest normalized Ct value: RQ=1.0) of a given mRNA of interest compared to other Day 1 samples), taking into account the amplification efficiencies (Pfaffl, 2001). Student's T-test was used to determine statistically significant differences between Day 1 and Day 5 samples using GraphPad Prism v 8.0.

Supplementary File 1 Table. mRNA qPCR primers

Gene name	Forward (5′ – 3′)	Reverse (5′ – 3′)	R^2	Amplification efficiency (%)
marco	CTATTGGTCCACGTGGTGTG	GTGGCACCATCTTCACCTTT	0.999	98.4
MHC II alpha chain	CAGGTGGACCAGGAACAATC	TGAAGGGCAGACTGGAGAAC	0.998	92.4
ef1a2 [a]	GCACAGTAACACCGAAACGA	ATGCCTCCGCACTTGTAGAT	0.994	92.6

[a] The primer sequences for this normalizer transcript were previously published in Katan et al. (2019).

Results

A significant increase in both *marco* and *MHC II* mRNA expression was observed in Day 5 HKLs compared to Day 1 HKLs (Supplementary File 1 Figure).

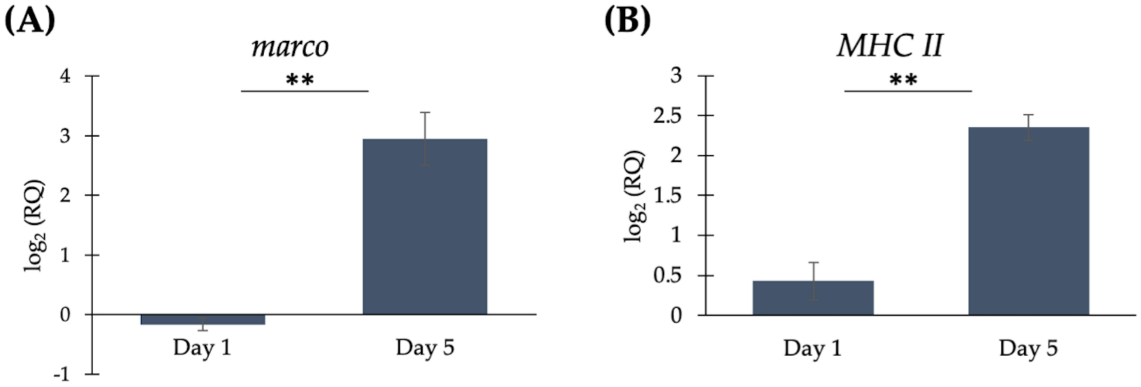

Supplementary File 1 Figure. qPCR confirmation of changes in macrophage gene expression in Day 1 and Day 5 HKLs. Data shown as mean $\log_2$ (RQ) ± s.e., n=3. ** indicates $p<0.01$, as determined by a paired Student's T-test. **A.** *marco*: macrophage receptor with collagenous structure **B.** *MHC II*: major histocompatibility complex II alpha chain

References

Katan, T.; Caballero-Solares, A.; Taylor, R.G.; Rise, M.L.; Parrish, C.C. Effect of plant-based diets with varying ratios of ω6 to ω3 fatty acids on growth performance, tissue composition, fatty acid biosynthesis and lipid-related gene expression in Atlantic salmon (*Salmo salar*). *Comparative Biochemistry and Physiology Part D: Genomics and Proteomics*. **2019**, 30, 290-304.

Pfaffl, M.W. A new mathematical model for relative quantification in real-time RT-PCR. *Nucleic Acids Res.* **2001**, 29, e45.
